# Spearmint Extract Containing Rosmarinic Acid Suppresses Amyloid Fibril Formation of Proteins Associated with Dementia

**DOI:** 10.3390/nu12113480

**Published:** 2020-11-13

**Authors:** Kenjirou Ogawa, Ayumi Ishii, Aimi Shindo, Kunihiro Hongo, Tomohiro Mizobata, Tetsuya Sogon, Yasushi Kawata

**Affiliations:** 1 Organization for Promotion of Tenure Track, University of Miyazaki, Miyazaki 889-2192, Japan; ogawa.kenjirou.u2@cc.miyazaki-u.ac.jp; 2 Department of Chemistry and Biotechnology, Graduate School of Engineering, Tottori University, Tottori 680-8552, Japan; bshijing80@gmail.com (A.I.); hongo@tottori-u.ac.jp (K.H.); mizobata@tottori-u.ac.jp (T.M.); 3 Department of Biomedical Science, Institute of Regenerative Medicine and Biofunction, Graduate School of Medical Science, Tottori University, Tottori 680-8552, Japan; a.3kmt10@gmail.com; 4 Center for Research on Green Sustainable Chemistry, Tottori University, Tottori 680-8552, Japan; 5 R&D Department, Wakasa Seikatsu Co. Ltd., 22 Naginataboko-cho, Shijo-Karasuma, Shimogyo-ku, Kyoto 600-8008, Japan; sogon@blueberryeye.co.jp

**Keywords:** spearmint, rosmarinic acid, polyphenol, amyloid fibril, amyloid beta, alpha-synuclein, Tau, dementia

## Abstract

Neurological dementias such as Alzheimer’s disease and Lewy body dementia are thought to be caused in part by the formation and deposition of characteristic insoluble fibrils of polypeptides such as amyloid beta (Aβ), Tau, and/or α-synuclein (αSyn). In this context, it is critical to suppress and remove such aggregates in order to prevent and/or delay the progression of dementia in these ailments. In this report, we investigated the effects of spearmint extract (SME) and rosmarinic acid (RA; the major component of SME) on the amyloid fibril formation reactions of αSyn, Aβ, and Tau proteins in vitro. SME or RA was added to soluble samples of each protein and the formation of fibrils was monitored by thioflavin T (ThioT) binding assays and transmission electron microscopy (TEM). We also evaluated whether preformed amyloid fibrils could be dissolved by the addition of RA. Our results reveal for the first time that SME and RA both suppress amyloid fibril formation, and that RA could disassemble preformed fibrils of αSyn, Aβ, and Tau into non-toxic species. Our results suggest that SME and RA may potentially suppress amyloid fibrils implicated in the progression of Alzheimer’s disease and Lewy body dementia in vivo, as well.

## 1. Introduction

Alzheimer’s disease, Lewy body dementia, and Parkinson’s disease are often caused by the formation of fibrillar aggregated proteins (amyloid fibrils) of amyloid beta (Aβ), Tau (identified in Alzheimer’s disease patients), and α-synuclein (αSyn; identified in cases of Lewy body dementia and Parkinson’s disease) [1,2,3,4,5,6]. During the formation of these protein fibrils, various soluble cyto-toxic oligomeric species are formed prior to the maturation of insoluble fibrils [7]. The deposition of Aβ, Tau and αSyn plaques, both inside and outside vital nerve cells, affects synaptic function and is associated with symptoms of dementia, which include onset of cognitive defects such as the impairment of learning and memorizing capabilities in the mouse and human brain [8], as well as various biological and neurochemical symptoms such as astrogliosis, neuronal dystrophy, and decline in acetylcholine levels [9]. Accordingly, it is important to suppress the aggregation of these proteins associated with dementia to prevent these symptoms.

**Figure 1:**
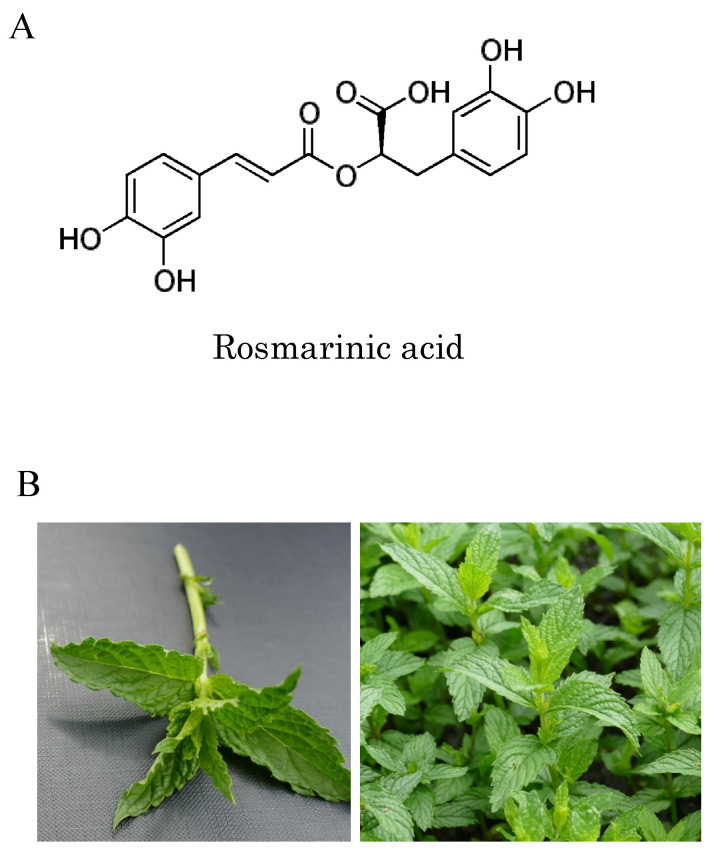
Chemical structure of rosmarinic acid and spearmint leaves: (**A**) chemical structure of rosmarinic acid; (**B**) spearmint (*Mentha spicata* L.) cultivated in Indiana, USA contains more rosmarinic acid and phenolic compounds than conventional spearmint due to selective breeding.

Spearmint (*Mentha spicata*) is a prominent member of the *Labiatae* family, which is noted for its high rosmarinic acid (RA) content [10] (the chemical structure of (R)-(+)-rosmarinic acid is shown in Figure 1A). A significant increase in RA content was achieved in cultivars produced in breeding experiments performed in Indiana, USA [11], and spearmint extracts (SME) used in food supplements are prepared from such specially cultivated species (shown in Figure 1B). In addition to a high concentration of RA, smaller amounts of 65 additional phenolic compounds may also be found in SME [12]. Administration of SME has been shown to prevent the degradation of cognitive functions such as learning and memory, and also to suppress the oxidation of brain tissue in senescence accelerated mouse-prone 8 (SAMP8) mice, a naturally occurring mouse line with an accelerated aging phenotype [13]. In addition, in human clinical trials, intake of SME by healthy elderly patients resulted in improvements in cognitive function, for example, attention span, concentration span, language comprehension abilities, and working memory [14,15].

Biochemically, RA has been shown to promote numerous biological activities, including antioxidative, anti-inflammatory, antiangiogenic, neuroprotective, antimicrobial, and immunomodulatory activities [16,17,18,19,20,21,22]. Previously, it was reported that RA displays the ability to prevent the oligomerization of αSyn, reduce the deposition of Aβ in mouse brains and suppress synaptic toxicity [20,23,24]. However, there are no reports regarding the in vitro effects of SME and RA upon the amyloid fibril formation/aggregation reactions of proteins such as Aβ peptide, Tau, and αSyn.

In the present study, we demonstrate that SME and RA are capable of directly suppressing the amyloid fibril formation of αSyn, Aβ, and Tau in vitro, by diverting molecules of these proteins toward a non-aggregated form. Furthermore, we show that RA is also capable of destabilizing and disassembling pre-formed amyloid fibrils of αSyn, Aβ, and Tau proteins. From these interesting results, we suggest that it may be feasible to achieve an effective suppression of the formation and the accumulation of amyloid fibrils related to dementia using SMA and RA.

## 2. Materials and Methods

### 2.1. Materials

SME samples were purchased from Kemin Japan Co. Ltd. (Tokyo, Japan), and the composition of compounds in the extracts was confirmed by high performance liquid chromatography (HPLC). Analysis showed that SME preparations typically consisted of mainly RA (12.0%), with trace amounts of various other phenolic components. Pure RA was purchased from FUJIFILM Wako Pure Chemical Co. Ltd (Osaka, Japan). Human Aβ_1-42_ peptide was purchased from Peptide Institute, Inc. (Osaka, Japan). Thioflavin T (ThioT) was obtained from Wako (Osaka).

### 2.2. Expression and Purification of αSyn and Tau Proteins

Human αSyn was over-expressed in *Escherichia coli* (*E. coli*) and purified according to methods reported previously [25]. For preparation of αSyn samples, lyophilized purified αSyn was dissolved in 4 M guanidine hydrochloride and then desalted with a PD-10 column (GE Healthcare, Tokyo, Japan). The concentration of αSyn in the samples was determined by using a molar absorption coefficient of ε 280 nm = 0.354 [26].

A pET23a-hTau40 gene was constructed by ligation of a synthesized hTau40 gene optimized for *E. coli* expression (Thermo Fisher, Waltham, MA, USA) with a DNA fragment obtained from the expression vector pET-23a(+). Both fragments were digested with the restriction enzymes *Nde*I and *Hin*dIII prior to ligation. After validation through DNA sequencing, the resultant pET23a-hTau40 expression vector was introduced into *E. coli* BLR(DE3) (Novagen) to establish an over-expression system for the human Tau protein (*E. coli* BLR(DE3)/pET23a-hTau40). The cultured cells were suspended in purification buffer (50 mM Tris-HCl, pH 7.8, containing 2 mM EDTA•2Na, 2 mM dithiothreitol (DTT), 0.2 mM phenylmethylsulfonyl fluoride (PMSF)) and incubated on ice before disruption using a combination of lysozyme chloride and sonication. After this process, the supernatant was recovered by centrifugation of the sample at 10,000 rpm, at 4 °C for 20 min. Sodium chloride was then added to the supernatant to a final concentration of 0.5 M, after which samples were heated to 80 °C for 10 min. Immediately afterwards, samples were cooled on ice, and then centrifuged to remove insoluble matter. Streptomycin sulfate (final concentration was 2.5%) was added to this clarified supernatant and the sample was stirred on ice for 30 min to precipitate nucleic acids. After removal of precipitated nucleic acids by centrifugation, the supernatant was dialyzed overnight against purification buffer. The dialysate containing Tau was centrifuged to remove debris and applied to an SP-Sepharose cation exchange column equilibrated with purification buffer. Bound samples were eluted by applying a linear gradient of 0–0.5 M NaCl. Eluted fractions containing Tau were recovered and dialyzed against 1 mM ammonium bicarbonate, and then lyophilized to obtain the final purified sample. Protein concentrations of purified Tau were determined by using a protein dye assay (Protein Assay Kit, Bio-Rad Laboratories) with bovine serum albumin (Sigma-Aldrich Japan, Tokyo, Japan) as a standard.

### 2.3. Amyloid Fibril Formation and ThioT Binding Assay

αSyn: αSyn (1 mg/mL) was incubated in 50 mM Tris-HCl buffer, pH 7.0, containing 150 mM NaCl, and 20 μM ThioT (Wako) with or without various concentrations of SME and RA added, in 96-well plates (Greiner, Kremsmuenster, Austria). Sample plates were incubated and monitored for changes in ThioT-derived fluorescence at 37 °C using an ARVO X (PerkinElmer Japan Co., Ltd., Yokohama, Japan) fluorescent plate reader with continuous agitation. ThioT fluorescence intensities were monitored by using an emission cutoff filter at >486 nm, with the excitation wavelength set to 450 nm.

Aβ: amyloid fibril formation of Aβ by using ThioT binding assay was performed according to methods reported previously, with some modifications [27]. Lyophilized human Aβ_1-42_ was dissolved in aqueous 0.02% ammonia to prepare a 500 μM Aβ stock solution. Samples of 16.65 μM Aβ were prepared from this stock in 5 mM phosphate buffered saline (PBS) buffer, pH 7.4, containing 150 mM NaCl, 20 μM ThioT, and various concentrations of SME or RA dissolved in DMSO. Fluorescence changes were monitored using 96-well plates at 37 °C using a SpectraMax M2e multi-mode fluorescence plate reader (Molecular Devices, Tokyo, Japan) without agitation. ThioT fluorescence was monitored at 480 nm with an excitation wavelength of 440 nm. 

Tau: hTau40 (0.5 mg/mL) was incubated in 25 mM Tris-HCl buffer, pH 7.4, 150 mM NaCl, 2 mM DTT, 5 μM heparin, 20 μM ThioT and containing various concentrations of SME or RA. ThioT fluorescence of samples in 96-well plates was monitored at 37 °C using an ARVO X (Perkin Elmer) fluorescence plate reader with continuous agitation. Fluorescence intensities at >486 nm were monitored by using an emission cutoff filter, with the excitation wavelength set to at 450 nm. Alternatively, emission measurements were taken at more precise wavelengths using the SpectraMax M2e multi-mode fluorescence plate reader (Molecular Devices, USA) with the emission wavelength set to 480 nm and the excitation wavelength set to 440 nm.

### 2.4. Transmission Electron Microscopy (TEM) Measurements of Fibril Samples

TEM measurements were performed on a JEOL-1400plus transmission electron microscope operating at 80 kV, as previously described [25]. Samples of αSyn, Aβ, or Tau protein incubated with or without SME or RA were diluted five-fold with water and applied to collodion-covered carbon mesh disks for 90 sec. Excess samples were blotted off and the sample disks were briefly rinsed by applying 5 μL of Milli-Q water followed by immediate blotting. Samples were stained by the application of a ten-fold diluted solution of EM Stainer (Nisshin EM Co., Ltd., Tokyo, Japan) to these washed samples for 30 sec followed by the blotting and air-drying of the completed sample.

### 2.5. Measurement of Cell Viability

The cell viability measurements were performed according to previous studies on the mouse neuroblastoma cell line Neuro2a (N2a), using a Tali™ Image-Based Cytometer (Thermo Fisher Scientific, Waltham, MA, USA) [28]. N2a cells were obtained from Public Health England. Cells were grown in Minimum Essential Medium (MEM, Thermo Fisher Scientific, Waltham, MA, USA) containing 10% fetal bovine serum, MEM non-essential amino acid solution (FUJIFILM Wako Pure Chemical Corporation, Osaka, Japan), 100 μM sodium pyruvate solution (FUJIFILM Wako Pure Chemical Corporation, Osaka, Japan), and 100 U/mL penicillin-streptomycin (Thermo Fisher Scientific, Waltham, MA, USA). Cell stock was seeded into 48 well plates and cultured at 37 °C with 5% CO_2_ until the cells in the wells reached 80–90% confluence. N2a cells were then incubated with samples of amyloid fibril (either 1 mg/mL αSyn, 500 μM Aβ, or 0.5 mg/mL Tau) that had been pretreated with or without RA for differing intervals (40 h for αSyn, 12 h for Aβ, and 48 h for Tau). The specific times of incubation for amyloid proteins with RA were adjusted to correspond to the time required for fibril disassembly, detected by the decrease in ThioT fluorescence intensity for each protein (30 h and 40 h for αSyn, 7.5 h and 12 h for Aβ, and 36 h and 48 h for Tau). After incubation, fibril samples were collected for assays. Furthermore, 70% ethanol was used as a positive toxicity control. After incubating the cells for 24 h with each fibril sample, the cells were washed with PBS, collected to form cell suspensions, and then incubated with 1 μM 3’,6’-di(*O*-acetyl)-2’,7’-bis[*N*,*N*-bis(carboxymethyl)aminomethyl] fluorescein tetra-acetoxymethyl ester (Calcein AM) and 400 nM ethidium homodimer-1 (EthD-1) (LIVE/DEAD™ Viability/Cytotoxicity Kit for mammalian cells, Thermo Fisher Scientific, Waltham, MA, USA) for 30 min in the dark. Subsequently, 25 μL of the N2a cell suspension was injected into Tali™ Cellular Analysis Slides (Thermo Fisher Scientific, Waltham, MA, USA), after which changes in EthD-1 fluorescence were monitored with the Tali™ Image-Based Cytometer to estimate the number of dead cells in the sample.

### 2.6. Statistical Analysis

Data are presented as means ± SEM. Statistical comparisons were made using one-way analysis of variance followed by Student’s *t*-test, Dunnett’s multiple comparison test, or Tukey–Kramer multiple comparison test. A value of *p* < 0.05 was considered statistically significant. 

## 3. Results

### 3.1. Suppression of the αSyn, Aβ and Tau Amyloid Fibril Formations by SME

**Figure 2:**
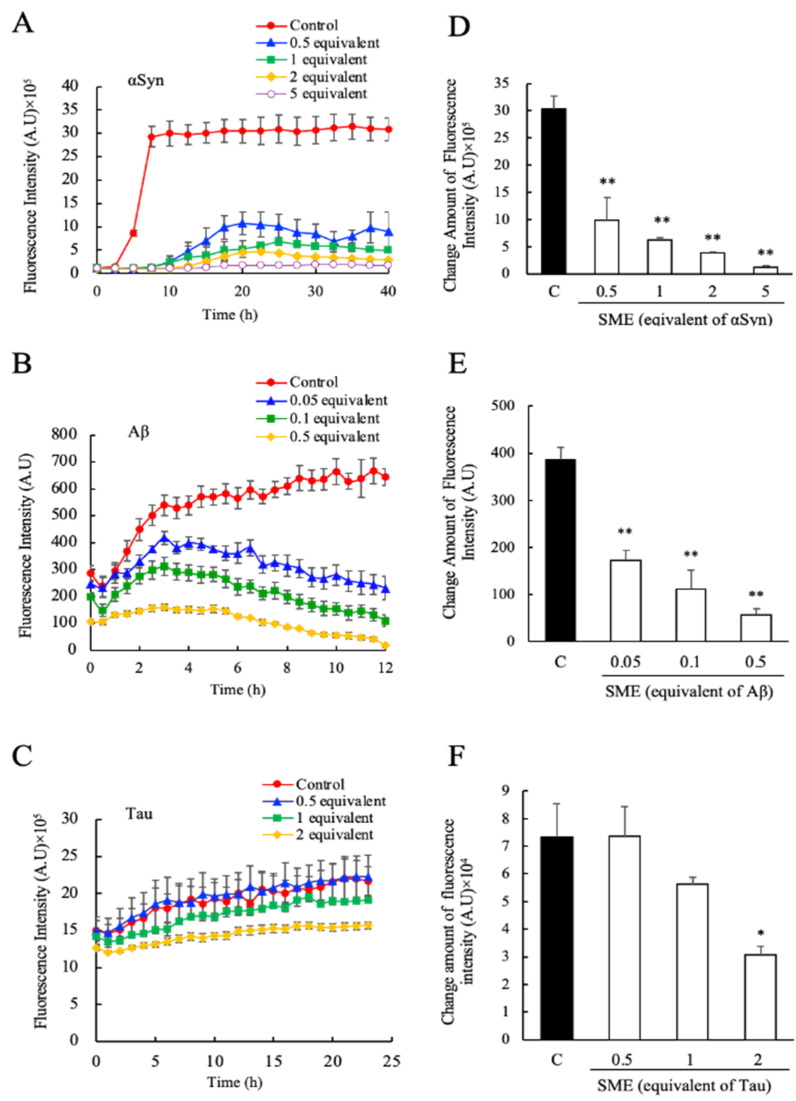
Inhibitory effects of spearmint extract (SME) against α-synuclein (αSyn), amyloid beta (Aβ), and Tau amyloid fibril formation. The degree of amyloid fibril formation was detected over time by measuring the specific fluorescence of fibril-bound thioflavin T (ThioT) in the presence and absence of SME; (**A**) αSyn (0.5–5 equivalents rosmarinic acid (RA)), (**B**) Aβ (0.05–0.5 equivalents RA), (**C**) Tau (0.5–2 equivalents RA). The net amount of fluorescence intensity change was estimated by the ratio between the maximum value and initial measurement value of its intensity; (**D**) αSyn, (**E**) Aβ, (**F**) Tau. Data are means ± SEM (*n* = 3). C, control. * *p* < 0.05, ** *p* < 0.01 vs. the control group (Dunnett’s multiple comparison test).

First, we examined the effects of direct SME addition to the fibril formation reactions of αSyn, Aβ, and Tau, in individual assays. The formation of amyloid fibrils of αSyn, Aβ, and Tau were monitored for 40 h, 12 h, and 25 h, respectively, by utilizing the specific fluorescence that is emitted by fibril bound ThioT. The concentrations of SME added to each sample were calculated to correspond to 0.5, 1, 2, and 5 molar equivalents of RA relative to αSyn (Figure 2A), 0.05, 0.1, and 0.5 molar equivalents of RA relative to Aβ (Figure 2B), and 0.5, 1, and 2 molar equivalents of RA relative to Tau (Figure 2C), respectively. In the absence of SME (shown as control), an increase in ThioT fluorescence intensity over time for each protein was observed, which reflected a typical fibrillation reaction time course of each protein. In the presence of SME, however, fibrillation was either completely suppressed or reduced significantly. Fibrillation of αSyn and Aβ was almost completely inhibited by the addition of SME at concentrations that corresponded to a five-fold molar equivalent of RA relative to the protein monomer. A prolongation of the lag-phase interval was also observed for αSyn (Figure 2A). In the case of Aβ samples containing SME, the ThioT fluorescence was seen to increase initially to a maximum value, and subsequently this intensity decreased gradually to almost the original values at the beginning of each experiment (Figure 2B). We plotted the ratio of maximum to minimum ThioT fluorescence intensities for each sample, as shown in Figure 2A–C, to gauge the concentration-dependent effects of SME on the fibril reaction of each sample. As shown in Figure 2D–F, significant decreases in the maximum/minimum ratio were observed in samples containing substoichiometric concentrations of RA (0.5 molar equivalent for αSyn, 0.05 molar equivalent for Aβ), and a more moderate but significant suppressive effect was seen when two-molar equivalents of RA were added to Tau, respectively. 

### 3.2. Suppression of the αSyn, Aβ and Tau Amyloid Fibril Formations by RA

**Figure 3:**
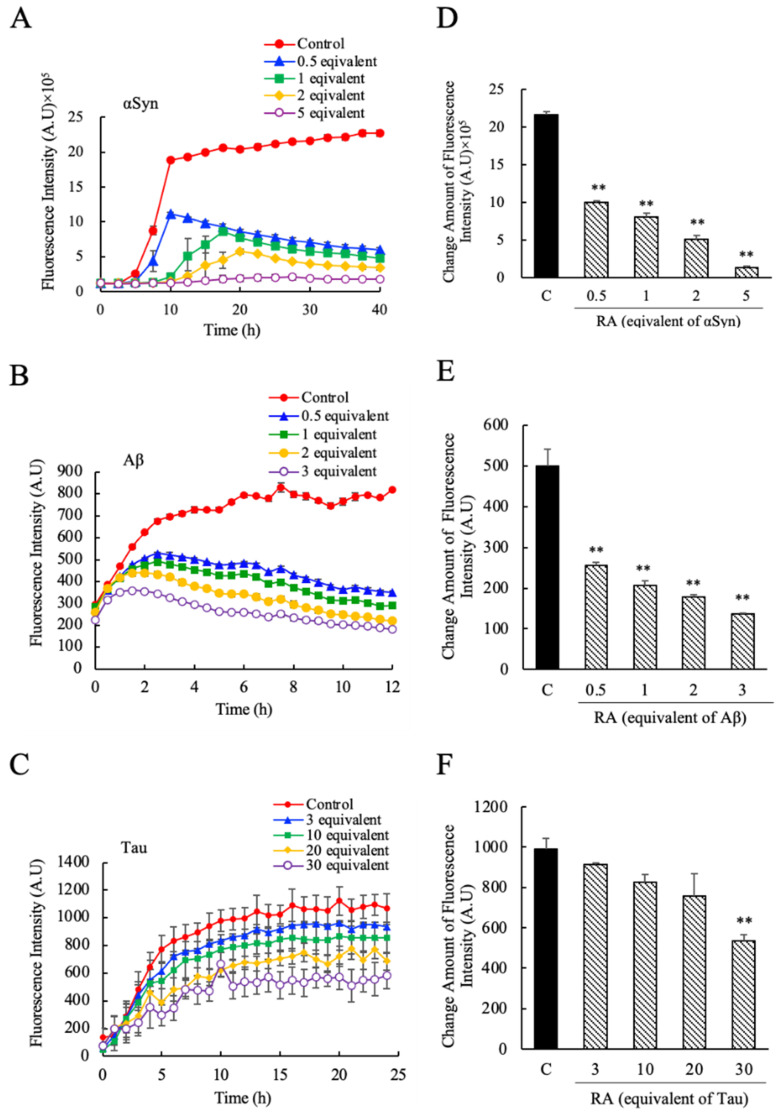
The inhibitory effects of pure rosmarinic acid (RA) against α-synuclein (αSyn), amyloid beta (Aβ), and Tau amyloid fibril formation. The degree of amyloid fibril formation was detected over time by measuring ThioT fluorescence in the presence and absence of pure RA; (**A**) αSyn (0.5–5 equivalent-mol RA), (**B**) Aβ (0.5–3 equivalent-mol RA), (**C**) Tau (3–30 equivalent-mol RA). The net amount of fluorescence intensity change was estimated by the ratio between the maximum value and initial measurement value of its intensity; (**D**) αSyn, (**E**) Aβ, (**F**) Tau. Data are means ± SEM (*n* = 3). C, control. ** *p* < 0.01 vs. the control group (Dunnett’s multiple comparison test).

Next, we evaluated the effects of pure RA on αSyn, Aβ, and Tau amyloid fibril formation. The concentrations of RA tested corresponded to 0.5, 1, 2, and 5 molar equivalents for αSyn, 0.5, 1, 2, and 3 molar equivalents for Aβ, and 3, 10, 20, and 30 molar equivalents for Tau, respectively. As shown in Figure 3, similar to the effects seen for SME, the addition of RA suppressed the increase in ThioT fluorescence over time for all three proteins relative to the control sample. The fibril suppression effects on αSyn (Figure 3A,D), Aβ (Figure 3B,E), and Tau (Figure 3C,F) were similar to the effects seen for SME (Figure 2), although the effective concentrations were different. Experiments for Tau using concentrations of RA that were comparable to those used for the other two targets (0.5, 1, 2 molar equivalents) failed to elicit a measurable effect on Tau fibrillation (data not shown), suggesting that the concentrations of pure RA required to alter Tau fibrillation were higher than the concentrations of SME needed to trigger a similar response. This unexpected result suggests that there may be an additional component present in SME that complements or enhances the effects of RA, which is relevant only to Tau fibrillation.

### 3.3. Suppression of Amyloid Fibril Formations of αSyn, Aβ, and Tau Detected by TEM

**Figure 4:**
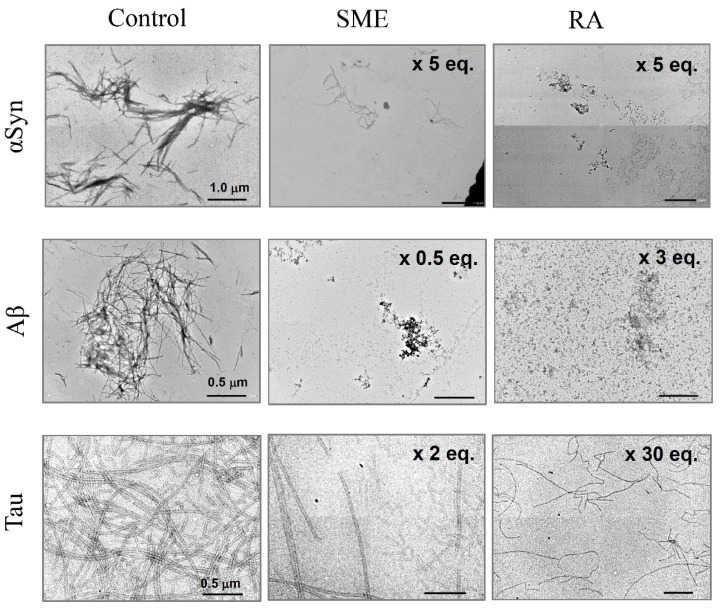
Transmission electron microscopy (TEM) measurements of amyloid fibrils of α-synuclein (αSyn), amyloid beta (Aβ), and Tau incubated with spearmint extract (SME) or pure rosmarinic acid (RA). αSyn, Aβ, and Tau amyloid fibril peptides were incubated with SME (5, 0.5, or 2 equivalent molar RA to each peptide) or pure RA (5, 3, 30 equivalent molar to each peptide) for the respective intervals (40 h for αSyn, 12 h for Aβ, and 25 h for Tau), after which samples were treated for observation with TEM. Scale bars, 1.0 μm on αSyn images, 0.5 μm on Aβ and Tau images.

From the results that we show in Figure 2 and Figure 3, we observe that both SME and RA have the ability to suppress amyloid fibril formation of αSyn, Aβ, and Tau. In order to determine the specific effects of SME and RA on aggregate morphology, we next observed samples of αSyn, Aβ, and Tau incubated in the presence and absence of SME and RA using TEM. Samples were taken from the end point of each fibrillation experiment shown in Figure 2 and Figure 3. As shown in Figure 4, the structure of amyloid fibrils formed by αSyn, Aβ, and Tau in the absence of SME or RA may be described as bundles of linear fibril structures (shown as control). In contrast, in samples of the three amyloidogenic proteins incubated with SME (five-fold molar equivalent to αSyn, 0.5-fold molar equivalent to Aβ, and two-fold molar equivalent to Tau) or RA (five-fold molar equivalent to αSyn, three-fold molar equivalent to Aβ, and 30-fold molar equivalent to Tau), visible fibrillar structures were markedly reduced. Additionally, extremely short fibril structures and amorphous, non-linear aggregates were both detected in samples incubated with SME or RA. In the case of the Tau protein, we could observe long fibrillar structures even in samples containing SME or RA, although the relative abundance of these structures in the samples was low. The fibrillar structures of Tau in the presence of RA also tended toward more twisted and curved structures, although we were unable to quantify these morphological differences. These results clearly show that SME and RA altered the morphologies of αSyn, Aβ, and Tau protein aggregates in a specific manner. 

### 3.4. Disassembly of Pre-Formed Amyloid Fibrils of αSyn, Aβ, and Tau by Addition of RA and Toxicity Evaluation

**Figure 5:**
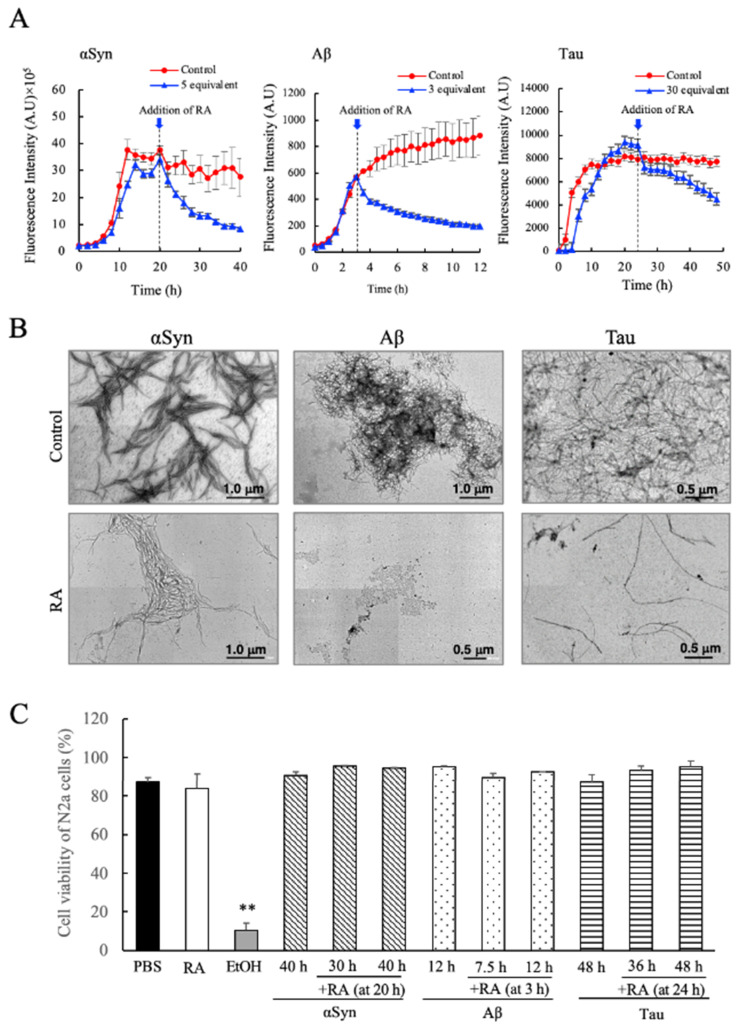
Evaluation of the fibril dissociative abilities of rosmarinic acid (RA) on preformed amyloid fibrils of α-synuclein (αSyn), amyloid beta (Aβ), and Tau. (**A**) RA was added to experiments where each fibrillogenic protein was allowed to form fibrils for a designated interval (20 h for αSyn, 3 h for Aβ, and 24 h for Tau). The specific instance that RA was added to each sample is denoted within each figure by the dotted lines and arrows. (**B**) TEM images of αSyn (at 40 h), Aβ (at 12 h), and Tau (at 48 h) samples after the addition of RA according to the protocol described in (**A**). (**C**) Determination of the cytotoxicity of RA-containing fibril samples, measured by Tali™ Cytometer analysis on Neuro2a (N2a) cells. Each fibril sample (either treated or not treated with RA) was collected at the indicated times (at 30 h and 40 h for αSyn, 12 h and 7.5 h for Aβ, 36 h and 48 h for Tau) and assayed for cytotoxicity. Data are means ± SEM (*n* = 3). PBS, phosphate buffered saline; EtOH, 70% ethanol solution. There are no significant differences in toxicity between RA-treated and non-treated fibril samples. ** *p* < 0.01 vs. the PBS groups (Tukey–Kramer multiple comparison test).

In the time course experiments shown in Figure 3A,B, we observed that for αSyn and Aβ, the ThioT fluorescence intensities in the presence of RA decreased after initially attaining a maximum value that was dependent on the concentration of added RA. From this result, we hypothesized that RA might possess the ability to disaggregate amyloid fibrils that had been formed. Therefore, in order to confirm this hypothesis, we studied the effects of adding RA to samples of preformed αSyn, Aβ, and Tau fibrils. As shown in Figure 5A, we observed that upon addition of RA to samples containing amyloid fibrils (as determined by the ThioT fluorescence intensities) a decrease in ThioT fluorescence was immediately triggered in each case. This fluorescence decrease seemed to be initiated regardless of the type of protein, and the effects of RA addition were independent of the specific stage at which RA was added to the reaction (for example, RA was able to trigger a fluorescence decrease during the extension phase of fibrillation of Aβ). TEM measurements confirmed that this decrease in ThioT fluorescence was accompanied by a loss of observable fibrillar aggregates (Figure 5B). These results clearly demonstrate that the RA can disassemble amyloid fibrils.

In the final experiment, we have evaluated the cytotoxicity of the disassembled species formed in the presence of RA using the Tali™ Image-Based Cytometer and the mouse N2a cell model. As shown in Figure 5C, we found that the disaggregated molecular samples that formed as a result of RA addition were completely inert against N2a cells, regardless of the type of fibrillogenic protein studied, or the time frame where RA addition was initiated. The result demonstrates rather clearly that RA disassembled amyloid fibrils of αSyn, Aβ, and Tau to a non-toxic, soluble form. In particular for Tau protein, this is the first instance of an isolated and characterized compound that shows such potent effects on fibril morphology and cytotoxicity. 

## 4. Discussion

In the present paper, we describe our efforts to characterize the suppressive effects of SME and its main component RA on the fibrillation of αSyn, Aβ, and Tau. SME is the water-soluble extract from a specially cultivated spearmint grown in Indiana, USA containing an enriched concentration of RA compared to other common cultivars. We demonstrated through our experiments that SME suppressed the in vitro amyloid fibril formation of αSyn, Aβ and also, for the first time, Tau protein (Figure 2 and Figure 4). We further determined that pure RA could also effectively suppress fibrillation (Figure 3 and Figure 4). This latter result strongly suggests that RA was the active component in SME that prevented the formation of characteristic amyloid fibrils of αSyn, Aβ, and Tau. Various polyphenolic compounds, including an antioxidative polyphenol RA [29], have been shown in previous studies to inhibit the formation of amyloid fibrils of proteins such as αSyn and Aβ [30,31]. Although RA has been shown to suppress the accumulation and aggregation of Aβ in vitro and in vivo [24,32], studies to determine whether SME (which contains a rich amount of RA) has the capability to directly suppress the fibrillation of proteins such as αSyn and Tau have not been performed to date.

From a detailed analysis of our data regarding the effects of SME and RA on fibril formation, we determined that the nature of the suppressive effects shown by these two chemical preparations was slightly different for each protein target. For instance, with respect to αSyn, SME and RA affected not only the rate of ThioT fluorescence increase (which reflects the rate at which fibrils are formed), but also affected the lag phase of fibrillation, which was prolonged (Figure 2A and Figure 3A). This suggested that SME and RA inhibited both initial fibril nucleus formation as well as fibril extension of αSyn. In contrast, an increase in the lag interval was not observed in experiments using Aβ and Tau. Interestingly, when we compared the concentration dependence of the suppressive effects of SME and RA on the three targets studied, we identified another subtle difference. In the case of αSyn, the concentration range in which the effect was observed was similar (was roughly the same order) for both SME and RA (0.5–5 equivalent molar; Figure 2A,D and Figure 3A,D). In contrast, with regard to Aβ and Tau, the effective concentration of SME that was required to bring about a suppressive effect was roughly 10-fold lower for SME compared to pure RA (Figure 2B,E and Figure 3B,E for Aβ; Figure 2C,F and Figure 3C,F for Tau). This interesting discrepancy suggests that SME is more potent in suppressing Aβ and Tau fibrillation compared to pure RA, and indicates that additional active compounds may be present in SME, and that we are observing in our experiments a differential effect of such additional compounds for αSyn versus the other two targets. The SME used in our experiments typically contains 65 additional minor components other than RA and its derivative, such as quinic acid, citric acid, caftaric acid, coumaric acid, salvianolic acid, coumaric acid, caffeic acid, ferulic acid, rutin (quercetin-rutinoside), luteolin, narirutin (naringnin-7-O-rutinoside), sagerinic acid, acacetin, apigenin, danshensu (dihydroxyphenyllactic acid), and their derivative hydroxylated forms [12]. These additional compounds may potentially bind to the proteins susceptible to forming amyloid fibrils and elicit an effect similar to that seen for RA [33,34,35]. It would be necessary to identify the effects derived from each phenolic composition included in SME, especially against Aβ and Tau, in future experiments to clarify this. 

One of the more intriguing results seen in our experiments was that SME displayed suppressive effects against Tau fibrillation in vitro (Figure 2C,F). Fibrillated Tau protein has been linked strongly to neurodegeneration, explained as the cause of disrupted axonal transport in Alzheimer’s disease and related tauopathies [36]. Thus, we consider it significant that a naturally derived extract (SME) could suppress Tau fibrillation and aggregation so strongly. Another intriguing finding was the ability shown by RA to disassemble previously formed amyloid fibrils of αSyn and Tau (Figure 5A,B). Additionally, we found out that the molecular intermediates formed during the disassembly of preformed fibrils showed no cytotoxicity to N2a (Figure 5C). Although similar reports using a variety of polyphenols including RA were performed to find compounds that could destabilize preformed αSyn fibrils [30,37,38,39], as far as we know, this is the first case where an evaluation of cell toxicity regarding the disassembled samples that were formed as a result have also been reported.

Finally, we would like to discuss potential molecular mechanisms by which SME and RA suppress and disassemble amyloid fibrils. Previous studies have suggested that the addition of certain compounds results in the chemical modification of proteins through interactions between polyphenol and certain amino acid side chains [40]. Chemical modification of Lys residues (at εNH_2_), by oxidized flavonoids derived from taxifolin or quercetin, was demonstrated to occur in experiments involving Aβ [41], and the significance of a non-covalent binding event of (-)-epigallocatechin gallate (EGCG) to the C-terminal region of αSyn was probed by NMR [42]. Although it was unknown whether covalent modification or non-covalent interaction occurs for Tau, it was reported that polyphenols, including catechol and flavonoids that have two adjacent phenolic hydroxyl groups, could effectively inhibit Tau filament formation [43]. These direct modification and non-covalent binding events of polyphenols to amyloidogenic proteins may result in the stabilization of the soluble monomer form of each protein, which would effectively stop the development of higher-order oligomers that eventually lead to the fibril form. Thus, it may be postulated that RA and SME (rich in RA) may function to suppress and to disassemble the fibrillation of αSyn, Aβ, and Tau proteins in a similar manner. It remains to be determined if a common molecular mechanism exists that explains the broadly applicable effects of RA and other potential polyphenols on protein fibrillation. Additionally, oral administration of SME to an experimental neurodegenerative disease animal model would be worthwhile in the future, since prevention of Alzheimer’s pathological Aβ aggregation and deposition in Tg2576 mice brain has been reported by oral administration of RA [44]. 

## 5. Conclusions

In conclusion, the present study suggests that water soluble SME and RA have the ability to suppress the amyloid fibril formation of αSyn, Aβ, and Tau, which is related to Lewy body dementia and Alzheimer’s disease. Furthermore, RA is able to disassemble preformed aggregated fibrils of these molecular targets into non-toxic species. In the future, we expect that functional research of SME will progress to clinical trials to elucidate the potential of spearmint to prevent dementia-related ailments.
